# Spatial Enablement to Support Environmental, Demographic, Socioeconomics, and Health Data Integration and Analysis for Big Cities: A Case Study With Asthma Hospitalizations in New York City

**DOI:** 10.3389/fmed.2019.00084

**Published:** 2019-04-24

**Authors:** Daniele Pala, José Pagán, Enea Parimbelli, Marica Teresa Rocca, Riccardo Bellazzi, Vittorio Casella

**Affiliations:** ^1^Department of Electrical, Computer and Biomedical Engineering, University of Pavia, Pavia, Italy; ^2^Department of Public Health Policy and Management, College of Global Public Health, New York University, New York, NY, United States; ^3^Telfer School of Management, University of Ottawa, Ottawa, ON, Canada; ^4^Department of Civil Engineering and Architecture, University of Pavia, Pavia, Italy

**Keywords:** public health, data integration, spatial enablement, geostatistics, asthma, clustering, regression

## Abstract

The percentage of the world's population living in urban areas is projected to increase in the next decades. Big cities are heterogeneous environments in which socioeconomic and environmental differences among the neighborhoods are often very pronounced. Each individual, during his/her life, is constantly subject to a mix of exposures that have an effect on their phenotype but are frequently difficult to identify, especially in an urban environment. Studying how the combination of environmental and socioeconomic factors which the population is exposed to influences pathological outcomes can help transforming public health from a reactive to a predictive system. Thanks to the application of state-of-the-art spatially enabled methods, patients can be stratified according to their characteristics and the geographical context they live in, optimizing healthcare processes and the reducing its costs. Some public health studies focusing specifically on urban areas have been conducted, but they usually consider a coarse spatial subdivision, as a consequence of scarce availability of well-integrated data regarding health and environmental exposure at a sufficient level of granularity to enable meaningful statistical analyses. In this paper, we present an application of highly fine-grained spatial resolution methods to New York City data. We investigated the link between asthma hospitalizations and a combination of air pollution and other environmental and socioeconomic factors. We first performed an explorative analysis using spatial clustering methods that shows that asthma is related to numerous factors whose level of influence varies considerably among neighborhoods. We then performed a Geographically Weighted Regression with different covariates and determined which environmental and socioeconomic factors can predict hospitalizations and how they vary throughout the city. These methods showed to be promising both for visualization and analysis of demographic and epidemiological urban dynamics, that can be used to organize targeted intervention and treatment policies to address the single citizens considering the factors he/she is exposed to. We found a link between asthma and several factors such as PM2.5, age, health insurance coverage, race, poverty, obesity, industrial areas, and recycling. This study has been conducted within the PULSE project, funded by the European Commission, briefly presented in this paper.

## Introduction

The percentage of the world's population living in urban areas is projected to increase from 54% in 2015 to 60% in 2030 and to 66% by 2050 (1). In absolute terms, more than 1 billion people were added to urban areas between 2000 and 2014. It is important to recognize that cities are not just economic drivers for countries, but also perfect labs for innovation and research aiming to manage and respond to dramatic demographic and epidemiological transitions (2). Big cities are heterogeneous environments where social and environmental conditions vary significantly within relatively small distances. Therefore, in order to improve well-being, health in the big cities has to be addressed at a neighborhood level (3). Participatory Urban Living for Sustainable Environments (PULSE) is an international project funded by the European Commission under the Horizon 2020 framework to undertake research and innovation in cities in Europe, the United States and Asia. The project started in late 2016 and has a planned total duration of 3 years. PULSE is partnering with municipality leaders of five major cities—Paris, Singapore, Birmingham, Barcelona, and New York—to collect information from the public health system, remote and fixed environmental sensors, and citizen-operated mobile devices, to develop a system for the management of public health policies in the urban environment, being first to build an integrated approach to public health challenges in cities.

PULSE has two main clinical focuses: the link between air pollution and the respiratory disease of asthma (4), and the one between physical inactivity and the metabolic disease of type 2 diabetes (5). Within PULSE, health risk is understood to be a combination of environmental and social exposures (e.g., air pollution, poverty) and human behavior (e.g., a sedentary lifestyle). The overall goal is to build an extensible platform and technologies to predict, mitigate, and manage public health problems, and promote community health and well-being, in cities (6). This goal is pursued by establishing an integrated data ecosystem based on continuous large-scale collection of heterogeneous data available within the smart city environment, in which both the citizens and the public health authorities are directly involved, the former through a personal smartphone app, the latter through dedicated visualization and simulation tools. Data is collected directly from the citizens that take part to the project and from a set of sensors and open sources.

The project will culminate with the establishment of Public Health Observatories (PHOs) in each urban locality, which will mainly rely on the analytics components of the PULSE system to integrate, analyze and visualize data to inform health policy decisions and support their evaluation in a data-driven fashion.

One of the key innovation aspects of this project, is the application of spatial enablement, i.e., the ability to add a geographic reference to data and information, to urban data analytics, in order to find important solution in a restricted but populated area such as a dense city.

In this article, in line with PULSE's target and the principles of its data integration approach, we present an application of spatially-enabled analytics to historical New York City data gathered in the context of the project, to investigate the possible links between air pollution, socioeconomic factors, and asthma hospitalizations. Within PULSE, an extensive research on asthma physiopathology and determinants has been carried out, integrating data and information from the literature that can allow a proper design of a prevention/treatment system that focuses on the most relevant factors, to properly inform citizens and public health operators, and provide them with tools that ease the intervention process.

Asthma is known to be a complex multifactorial disease, whose manifestation and exacerbation is related to a combination of genetic, social and environmental factors. Some of the mechanisms that define how these factors influence the disease are known to the scientific community (7), for instance the relation between air pollution and asthma exacerbation (8) and the connection between asthma prevalence and demographic or socioeconomics conditions such as race, education, sex, and income (9). Despite this knowledge, studies that address the problem at an intra-city level, clustering neighborhoods and population according to their risk level and allowing policy makers to better target their interventions, are generally missing.

Modern research in medicine is exploring new ways to optimize healthcare services, creating approaches that are developed specifically for each patient taking into account his/her genetic and behavioral features and the environmental factors he/she is exposed to. This approach, commonly called Precision Medicine, allows to maximize the effectiveness of treatments in terms of benefits and time and resources spent. This objective is achieved using grouping tools that stratify similar patients in clusters according to certain characteristics, in order to find common patterns that can help design the best treatment for each patient belonging to a specific cluster.

Furthermore, recent research found that besides genetic predispositions, disease outcomes and phenotype in general is influenced by what is usually called *exposomics*, i.e., a combination of exposures to multiple factors which an individual is subject to during a certain period of time (10). Spatial enablement can add an important contribution to public health management, citizens' well-being and precision medicine, since the geographic dimension cannot be neglected when it comes to considering environmental and socioeconomic exposures.

Using spatial enablement to carry out geospatial analysis of determinants of a complex disease such as asthma can be useful to study the problem at a neighborhood level, in order to find the right geographical and environmental exposures patterns that can determine which groups in the population are mostly at risk. This approach leads to better results than studying the problem at a single patient level, that would require longer experimentation times at the expense of healthcare resources and patients' health; nonetheless studying these relations considering the whole city with a-spatial methods would hide most of the important geo-dependent exposure aspects of the disease.

We therefore considered how both the environmental and social conditions vary among different city areas and how these factors impact on asthma outcomes throughout the different neighborhoods. Studying the way environmental and socioeconomic factors can determine the risk of asthma complications can be complicated in big cities, where millions of people live concentrated in a small space and environmental and social disparities are emphasized. A few studies focusing specifically on urban areas have been conducted, but they usually consider a coarse spatial subdivision which often corresponds to the whole city (11) or macroscopic grid-like subdivisions in the order of 10 × 10 km (12). This is often a consequence of the scarce availability of well-integrated data regarding health and environmental exposure at a sufficient level of granularity to enable meaningful statistical analyses.

In the final phase of PULSE, in which the PHOs will be functioning and aiding the public health decision making process, health authorities will be able to inspect aggregated data about the population merged with environmental data, and they will be provided with simulation tools that will allow monitoring of several disease outcomes based on social, environmental and behavioral factors. The asthma hospitalization rate is an example of outcome that is easy to monitor and useful to model, as public health authorities are interested in reducing hospitalizations rate for several reasons, that include improving the population's well-being and reducing healthcare costs and consumption of resources; this is particularly true in New York City, since the New York State healthcare system spends 1.3 Billion dollars per year for asthma (second highest state in the US) and current asthma prevalence in adults living in New York City is 10.2%, higher than 9.3% of adults who live in the rest of the State (13).

This paper reports an initial exploration of some of the high spatial resolution methods that will be used to analyze the large heterogeneous data gathered in PULSE, demonstrating their necessity and usefulness. In this study, we used state of the art spatially-enabled analytics applied to data gathered in the context of PULSE regarding the urban area of New York City, subdivided into 42 sectors according to the official United Funded Hospital (UHF42) subdivision. In particular, we applied some spatial clustering methods and performed geographically weighted regression (GWR) using the 2014 asthma hospitalization rate as dependent variable and testing several covariates. Our results confirmed that the hospitalization rate is related to a number of environmental and socioeconomic factors whose level of influence changes in the different areas of the city. The application of spatially-enabled methods allowed us to discover important differences in the effect of the diverse factors in the different neighborhood of the city, none of which would be visible in studying the effect of the same factors for the whole city or for the main 5 administrative areas (Boroughs).

## Materials and Methods

### Data Sources

Several sources of data have been used to carry out the analyses reported in this paper. Most of the data has been kindly provided to the PULSE consortium by The New York Academy of Medicine, whereas other data has been gathered directly from open repositories. In particular, during the first year of the project, data from all the cities taking part at the project regarding air pollution, asthma prevalence, hospitalizations and ED visits, and several socioeconomic factors was gathered and integrated in the PULSE data repositories. The data to be collected was chosen based on the elements that appeared to be most related to asthma exacerbation according to previous evidence in literature. Although a large quantity of data is being collected within PULSE, open data has been gathered too where available. One of the cities with the largest availability of recent data is New York, that has been chosen for the preliminary study on the link between air pollution and asthma, and on the role of socioeconomic conditions in asthma-related visits to the hospital, reported in this paper. Several variables, listed and described in Table 1, have been chosen for this study. For air pollution, we chose the PM2.5 and ozone concentrations, as among all the pollutants they appear to be particularly relevant in asthma exacerbation (14, 15) and open data about their monitoring in NYC is easily available. We then chose a set of other environmental data related to asthma (e.g., industrial land use) and some socioeconomic factors that appeared to be relevant in previous research (8, 12). See Table 1 for details.

**Table 1:** Description of the data used in our study.

**Type**	**Description**	**Source**	**Year**	**Sample size**
Health-related	Hospitalization rate: number of people hospitalized for asthma over total population	SPARCS, NYC Data Portal	2014	42 observations (one for each UHF42)
Environmental	PM2.5 yearly average	NYC Data Portal	2014	42 observations (one for each UHF42)
Environmental	Ozone summer average (from June to September)	NYC Data Portal	2014	42 observations (one for each UHF42)
Environmental	Percentage of land used for industrial activities	Data2go.nyc	2017	59 observations (one for each CD59)−42 after interpolation
Environmental	Recycling rate	Data2go.nyc	2010	59 observations (one for each CD59)−42 after interpolation
Demographic	Age: percentage of population aged < 18, average age at hospitalization and percentage of population aged >65	SPARCS	2014	174 observations (one for each zip code) for average age−42 (one for each UHF42) after interpolation. 42 observations for the percentages
Demographic	Race: percentage of people identifying as Black, Hispanic, Asian, White, Other/Unknown	NTA	2014	195 observations (one for each NTA)−42 (one for each UHF42) after interpolation
Socioeconomic	Poverty rate	NTA	2014	195 observations (one for each NTA)−42 (one for each UHF42) after interpolation
Socioeconomic	Medicaid coverage	NTA	2014	195 observations (one for each NTA)−42 (one for each UHF42) after interpolation

The socioeconomic data used in this paper is freely available in the NYC Neighborhood Health Atlas website (16), from which it has been downloaded. The hospitalization and ED visit rates data, as well as the PM2.5 historical data, has been downloaded from the NYC Environmental & Health Data Portal (17). The data regarding the percentage of land used for industrial purposes, the obesity rate and the recycling rate were taken from the data2go.nyc website (18). The information regarding age and race of hospitalized people has been acquired from the SPARCS (19) limited 2014 dataset.

### Data Integration

Public health and environmental data is usually collected with respect to different polygonal subdivisions, in New York. The most used ones are: UHF34 and UHF42 (United Hospital Fund with 34 and 42 polygons, respectively), CD55, CD59 and CD71 (Community Districts with 55, 59 and 71 polygons), NTA (Neighborhood Tabulation Areas) and ZIP (ZIP codes). In the present paper, we decided to adopt UHF42 to visualize the results, as most of the data considered is natively available for it. To overlap other layers with a different spatial subdivision, we applied the following data harmonization algorithm: let's consider a polygonal subdivision for which a certain variant is available, for each polygon $P_{i}$, the variant value *v*_*i*_ is known. If we consider another polygon $P_{0}$, belonging a different subdivision, in general it won't coincide with any $P_{i}$ and, instead, will overlap to several of them. The estimated *v*_0_ can be obtained by the weighted sum

(1) $$v_{0}=\frac{\sum_{i}v_{i}A_{i}}{\sum_{i}A_{i}}$$

where *A*_*i*_ is the area of the intersection between $P_{i}$ and $P_{0}$ and the summation is only performed over the polygons for which the intersection is non-empty. This means that the value of a certain phenomenon in any spatial subdivision can be represented in the UHF42 one constructing each polygon as the sum of the same value in the other subdivision, weighted for the overlapping area.

### Spatial Analysis

To perform our spatial clustering analyses we used the ArcGIS *Grouping analysis* tool (20), part of the *Spatial Analyst toolbox*. With this tool, given a geographic feature set as input, it's possible to find a certain number of subgroups such that the intra-group feature similarity and the inter-group feature dissimilarity are jointly maximized. This tool implements a clustering method based on Minimum Spanning Trees (21): a minimum spanning tree is devised that summarizes both feature spatial relationships and feature data similarity. Features become nodes in the minimum spanning tree connected by weighted edges. The weight for each edge is proportional to the similarity of the objects it connects. After building the minimum spanning tree, a branch (edge) in the tree is pruned, creating two minimum spanning trees. The edge to be pruned is selected so that it minimizes dissimilarity in the resultant groups, while avoiding (if possible) singletons (groups with only one feature). The Euclidean Distance has been used as distance measure between neighboring features. We set up a spatial constraint option that renders polygons that share at least one side/vertex to be more likely to be clustered together, thus reinforcing the analysis spatial component. The number of clusters has been automatically chosen using the Calinski-Harabasz pseudo F-statistic in order to better optimize the clustering.

The considered datasets were then analyzed by Geographically Weighted Regression (GWR) (22), whose basics are given here. Standard or *a-spatial* linear regression is based on the relation

(2) $$y=\beta_{0}+\beta_{1}x+\varepsilon$$

between the dependent variable *y* and the explanatory one, *x* in the simplest case, having only one explanatory variable. The variable *x* and *y* must be known for an adequate number *n* of observations, which can be represented by points (*x*_*i*_, *y*_*i*_). The thus-obtained set of equations can be represented in the vector formalism

(3) Y=Xβ+ε

where **Y** contains the actual measurements of *y*, ****ε**** is a vector with the error terms and the vector **β** is composed by the two unknowns, β_0_ and β_1_. Its estimation is usually performed by a minimum problem; the minimized quantity is the squared norm of the difference between the observed values of **Y** and those given by the model **Y**(****β****) = **Xβ**; in formula

(4) $$\hat{\beta }:\text{​​ }=\text{min}{\sum \left(y_{i}-{\left(X\beta \right)}_{i}\right)}^{2}$$

The depicted solution can be generalized by introducing a weight, thus giving each observation a different relevance

(5) $$\hat{\beta }:\text{​​ }=\text{min}{\sum w_{i}\left(y_{i}-{\left(X\beta \right)}_{i}\right)}^{2}$$

GWR uses the above defined method to take into consideration spatial variability. In a common GIS layer with polygon representation of an environment, each polygon corresponds to an observation, located in its *centroid*. The studied area can be overlapped with a set of regularly-spaced dots. For each dot, a distinct regression is calculated, in which the observed values for the dependent and explanatory variables are the same, but the weights change. In our case, the weight function is

(6) $$w_{i}=e^{-\frac{d_{i}^{2}}{s^{2}}}$$

where *d*_*i*_ is the distance between the considered dot and the *i*-th centroid and *s* is a threshold, corresponding in our case to 5 km. In our study, we overlapped to the NYC map a grid of points distant 1 km from each other. Due to the use of the UHF 42 subdivision, each univariate regression in this paper has 42 real observations (one for each centroid) overlapped with a grid of 48 × 48 points, for each one of which a different weight is assigned depending on the values of the closest centroids.

Other a-spatial methods have been used during this study to complete our analyses and improve the observation and explanations of the results. These methods are the standard Linear Regression (23), the Forward Stepwise Regression (24) and the One-Way ANOVA variance test (25) and they are not presented in this paper since we assume that the reader already knows their fundamentals.

## Results

In this section, we present the main results of the analyses that have been conducted on the data gathered within the context of PULSE regarding the city of New York.

**Figure 1:**
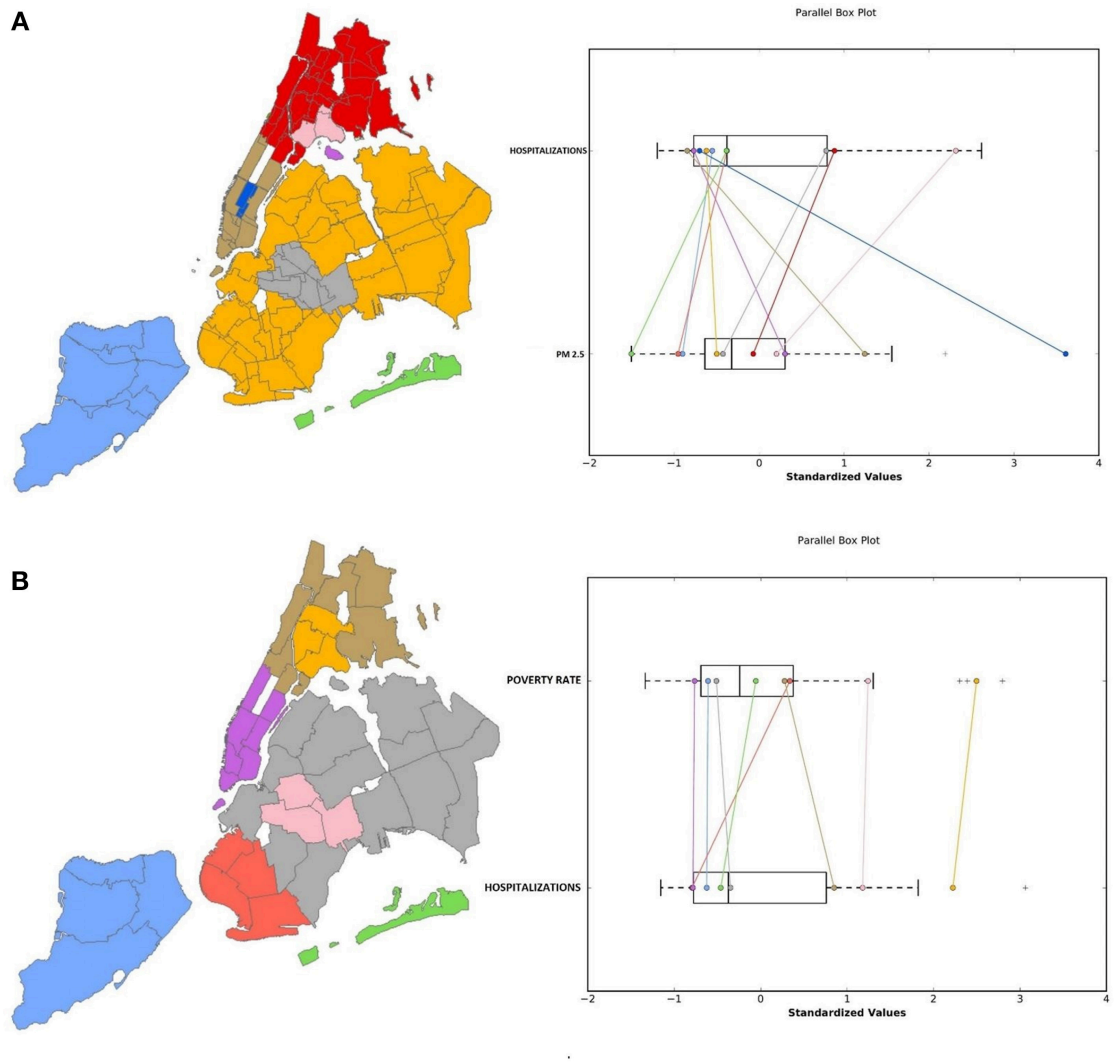
Results of the spatial clustering considering **(A)** the average 2014 PM2.5 concentration and the asthma hospitalization rate and **(B)** the poverty rate. On the left, a color-coded map of the clusters and on the right a parallel box plot that shows the relation between the parameters' distributions.

The first step of our exploratory analyses consisted in a set of spatially-enabled clustering analyses. In particular, in order to assess the relationship between air pollution and asthma hospitalizations, we first applied a spatial clustering method considering the rate of asthma hospitalizations and the yearly PM2.5 concentration as features. We performed the clustering multiple times, each one considering the data of a specific year from 2012 to 2014, obtaining systematically the same clusters as those shown in Figure 1A, showing 2014 data. Some interesting phenomena can be noticed in the clustering results:

- In 7 out of 10 clusters found by the algorithm, an occurrence of low PM2.5 concentration corresponds to a low hospitalization rate and vice versa;
- 3 out of 10 clusters, all within the borough of Manhattan, are in contrast with this tendency, since they present the highest PM2.5 levels together with the lowest hospitalization rates of all the city
- The Bronx, East Harlem and some neighborhoods in North/Central Brooklyn close to some of the biggest highways of the city (e.g., Brooklyn-Queens Expressway, Long Island Expressway) have the highest hospitalization rates of the whole city, together with the highest pollution level of all the neighborhoods excluding the wealthiest areas of Manhattan.

These results suggest that high air pollution levels are not the only factor that influences asthma hospitalization rate, indicating that socioeconomic factors play an important role, given that Manhattan, arguably the highest income area of the city, has jointly the highest air pollution levels and the lowest hospitalization rates. Previous research (8, 12) found significant correlation between asthma and socioeconomic factors like age, sex, education, race, and income. For these reasons, we ran additional analyses to investigate the spatial distribution of clusters of these variables and their potential association with asthma hospitalizations in the urban context. In particular, we used the open data from the New York Neighborhood Health Atlas project, that makes health and socioeconomic data categorized according to 195 Neighborhood Tabulation Areas (NTA) available. This data included: percentage of female population, percentage of population in the age ranges 0–18, 18–24, 25–44, 45–64, 65+, percentage of the population identifying as White, Black, Hispanic, Asian and other race, poverty rate, mortality rate and percentage of population with an education level under high school. Data was collected in 2014 and spatially categorized according to the New York 195 NTAs. In order to determine which of these variables were significant in influencing the hospitalization rate, we performed a forward stepwise regression, using the 2014 asthma hospitalization rate as the dependent variable. All the considered variables, excluding part of the race ones (percentage of Asians and races other than White, Black and Hispanic) and mortality rate, were significant.

Interesting results emerged from a subset of our clustering analyses where we focused on the link between child asthma hospitalizations and poverty. Figure 1B presents the results from the spatial clustering analysis using asthma hospitalizations and poverty rate. Excluding some neighborhoods in West Brooklyn, there seems to be an association between high poverty rates and high hospitalization rates. In particular, it is noticeable that in the same areas where the hospitalization rate is high (i.e., south Bronx and north/central Brooklyn), the poverty rate also appears to be higher than the rest of the city. We also examined the relationship between access to health insurance and asthma hospitalizations, since evidence suggests that people with Medicaid or who are uninsured use the ED more frequently than patients with private insurance (26). The clustering results show that the same areas that were found to have the highest hospitalization rates and the highest poverty rate (i.e., the Bronx, East Harlem and the neighborhoods close to the largest highways of Brooklyn and Queens) have the lowest percentage of insured people in the city. The situation is the opposite in most of Manhattan, Staten Island and the Rockaway peninsula.

Following preliminary exploratory analyses conducted using spatial clustering techniques, we performed a multivariate regression analysis using both standard and spatially-enabled linear regression.

Our results confirm the same phenomena pointed out by the spatial clustering (see Figure 1): we first trained a standard regression model using the 2014 hospitalization rate as dependent variable and the PM2.5 levels from the same year as covariate, and the results show that the PM levels do not have a significant impact on the hospitalization rate (*P*-value 0.985, Correlation Coefficient 0.003). Considering the spatial clustering results, in which it's clear that in midtown Manhattan the relationship between air quality and hospitalizations is different than the rest of the city, we carried out the same linear regression excluding all the neighborhoods of Manhattan but East Harlem, Central Harlem and Washington Heights (since they don't show the same contrast between high pollution and low hospitalization rate), and we obtained a significant relationship between air pollution and hospitalizations with a moderate positive correlation (*P*-Value 9.62 × 10^−4^, Correlation Coefficient 0.534). Using as covariate the 2014 poverty rate to predict the asthma hospitalizations, results show a very strong correlation (*P*-Value 5.93 × 10^−13^, Correlation Coefficient 0.855). From these results it could be assumed that the average yearly value of PM2.5 is not a good measure to study the effects of air quality on asthma, as local and brief but potentially dangerous peaks are not visible. Furthermore, these results confirm that, even if air quality plays a role in determining the number of asthma hospitalizations in several areas of the city, it appears that socioeconomic factors are much more decisive when all the city is considered. Hence, the spatial dimension cannot be neglected in studying the hospitalization rates in a city as NYC, in which there are pronounced socioeconomic and environmental differences among the neighborhoods. For these reasons, further analyses employing a Geographically-Weighted Regression (GWR, see Methods section for further details) were performed in order to correct for the local effect of socioeconomic factors in the different areas of the city. We applied our algorithm several times with a radius of 5 km and tested the different covariates, specifically: PM2.5 and ozone concentration in the same year (2014), poverty rate, percentage of the population identifying as Black, obesity rate, percentage of population aged under 18 or over 65, recycling rate.

### Air Pollution

**Figure 2:**
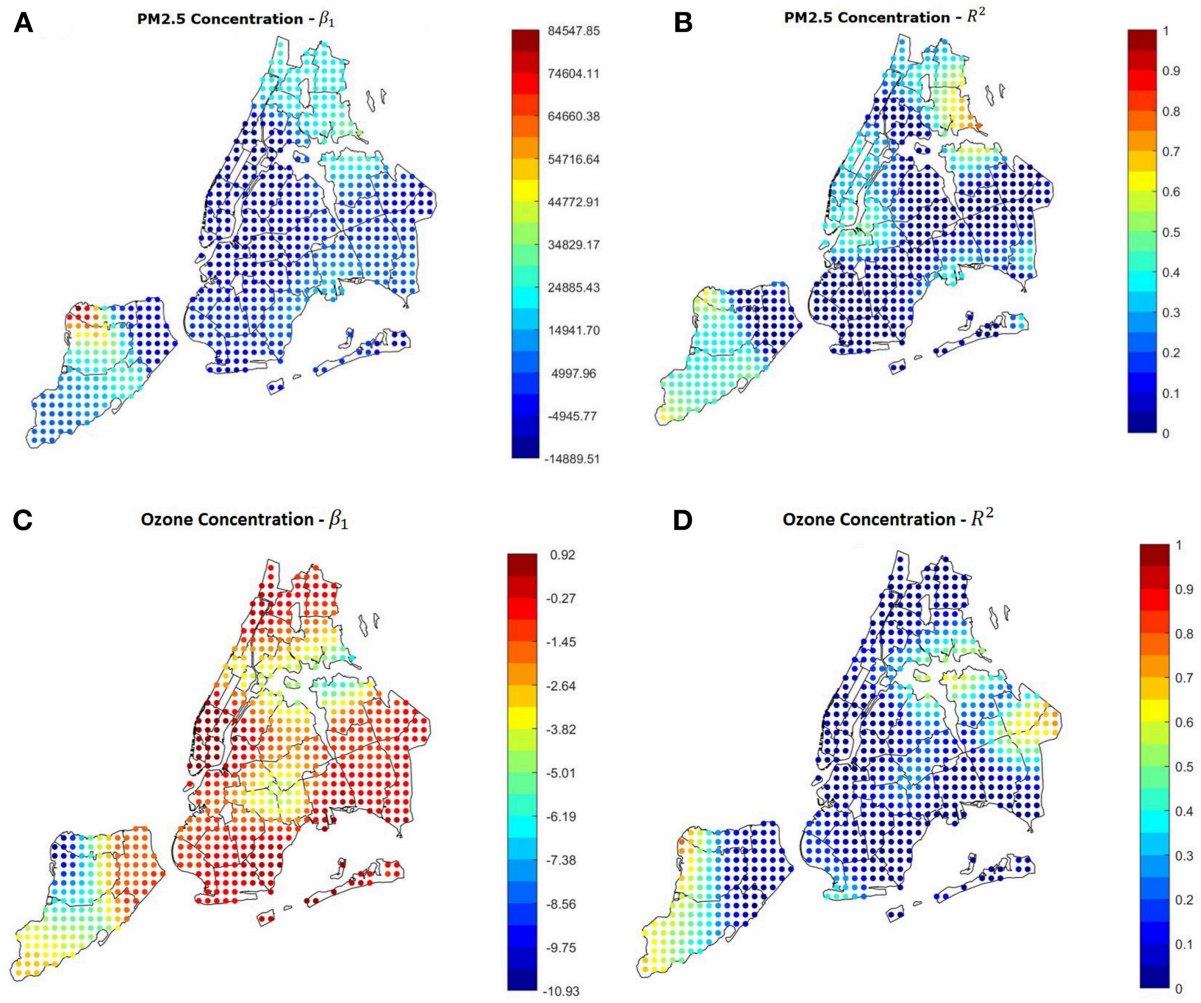
**(A,B)** Results of the GWR using the average yearly PM2.5 concentration as covariate. The yearly value is not a good predictor in most of the city. **(C,D)** Results of the GWR using the ozone summer concentration as covariate and the summer hospitalization rate as dependent variable. The relation is misleading in most of the city, indicating.

As shown in the previous section, according to our analyses air pollution, in particular the PM2.5 concentration, doesn't seem to have a significative impact on the asthma hospitalization rate in the whole city. Our results from the GWR confirm this hypothesis, since *R*^2^ is low in most of the city, and in Manhattan β_1_ is even negative, indicating the contrast between the high pollution level and the low hospitalization rate. Since asthma is known to be related to several pollutants, we applied the GWR also to the average concentration of Ozone. Since the open data about ozone available is only referred to summer months, we selected as dependent variable a subset of hospitalizations occurred from June to September 2014. Results are misleading and not reliable, since in most of the city *R*^2^ is low, and β_1_is generally negative. The overall correlation is−0.2186 and the *P*-Value is 0.164. This could be due to (i) higher influence of socioeconomic conditions than air pollution in the hospitalizations rate, (ii) low significance of averaged pollution data of a long period, that hides peaks and daily variations that can have an important effect on asthma. The GWR results regarding this part are shown in Figure 2.

### Race

**Figure 3:**
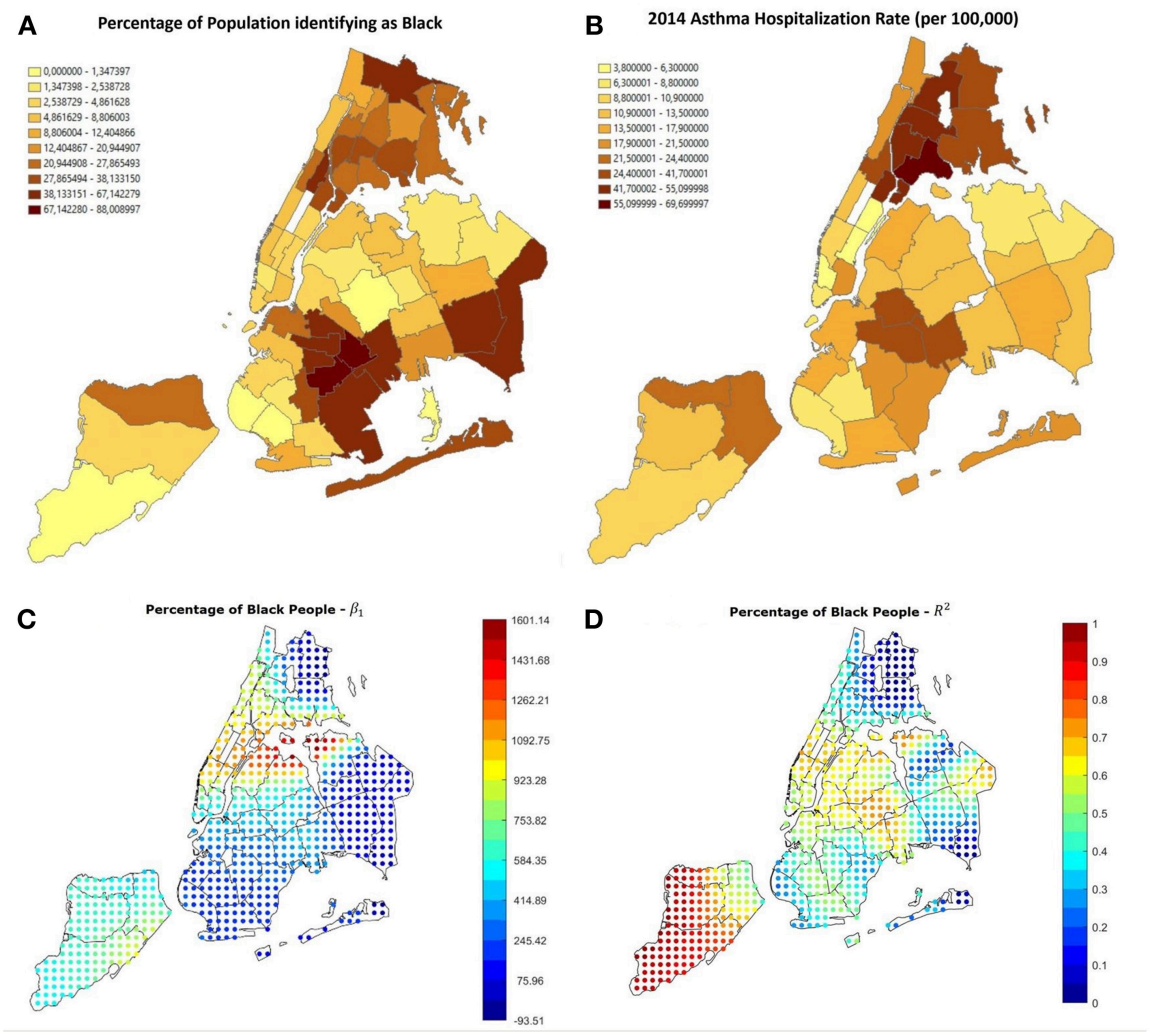
**(A)** Percentage of black people in the 42 districts in NYC. The highest numbers can be seen in the Bronx, in Harlem and East Harlem, South-East Queens and East Brooklyn, especially Crown Heights, Flatbush and Brownsville. Most of these areas are the same in which the hospitalization rate is high, visible in **(B)**. **(C,D)** GWR results using the percentage of people identifying as black as covariate. The correlation is positive and reliable in most of the city, especially all Manhattan, the Brooklyn-Queens border, Staten Island and South Bronx.

Several previous studies (27) suggested that race and ethnicity can be related to asthma risk and development. This is partially confirmed by our stepwise regression, that found a relation between asthma hospitalizations and percentage of Black, Hispanic, and White population. Table 2 shows the results of a first explorative analysis on the hospitalization rates per race in the five boroughs: each value is calculated as the number of hospitalizations divided by the number of people identifying as the specific race considered. Several things can be noticed: in the Bronx, Brooklyn and Queens, where the overall asthma rate is the highest, the hospitalization rate is higher for Black people. Previous studies (28, 29) suggested that in several areas of the USA Black people and Latinos tend to be more easily exposed to damaging pollutants since they usually live in areas close to industrial facilities and large highways. Figure 3 shows that the higher concentration of Black people in NYC takes place in the same areas where the higher hospitalization rates are. This phenomenon is mostly confirmed by the results of the GWR applied with the percentage of Black people as covariate, the results of which are visible in **Figures 5C,D**. The overall P-Value is 4.94 × 10^−4^ and the correlation coefficient is 0.5143.

**Table 2:** Percentage of people belonging to each race that was hospitalized in 2014 in each of the 5 Boroughs.

**Borough**	**Black**	**Hispanic**	**White**	**Asian**	**Other**
Bronx	0.0213	0.0015	0.0011	0.0008	0.031
Brooklyn	0.5951	0.1520	0.5606	0.1043	0.6203
Manhattan	0.0149	0.01	0.0144	0.0017	0.0515
Queens	1.3909	0.3003	0.6689	0.0287	0.4692
Staten Island	0.069	0.006	0.0026	0.001	0.0211

### Poverty Rate

**Figure 4:**
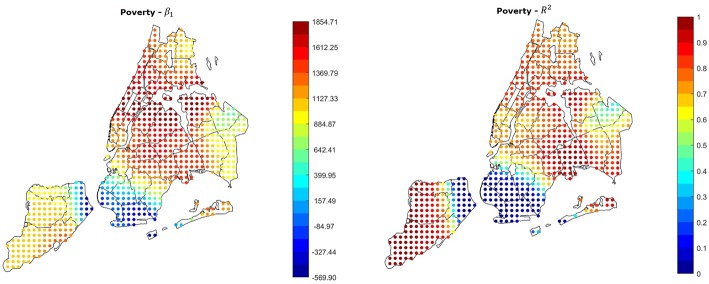
GWR results using the poverty rate as covariate. The relation is strongly positive and reliable in most of the city, the only exception is an area between South-west Brooklyn and East Staten Island.

As shown in the previous section, air pollution in NYC has a limited effect on the number of asthma hospitalizations in the city, whereas the poverty rate has a high correlation with it (*P*-Value 5.93 × 10^−13^, Correlation Coefficient 0.855). To investigate if and how this correlation varies throughout the city, we applied GWR also to the 2014 poverty rate in the different neighborhoods. Figure 4 shows the β_1_ and *R*^2^ parameters in the different areas of the city. It is noticeable that in most of the city β_1_ is positive and *R*^2^ has values > 0.5. This means that in most of the city the probability of observing asthma attacks increases with the poverty rate. In contrast with this, an area between south-west Brooklyn and east Staten Island shows values of *R*^2^ close to 0, meaning that in those neighborhoods the relation found is not reliable, and further analyses on those neighborhoods are required.

### The Effect of Age

**Figure 5:**
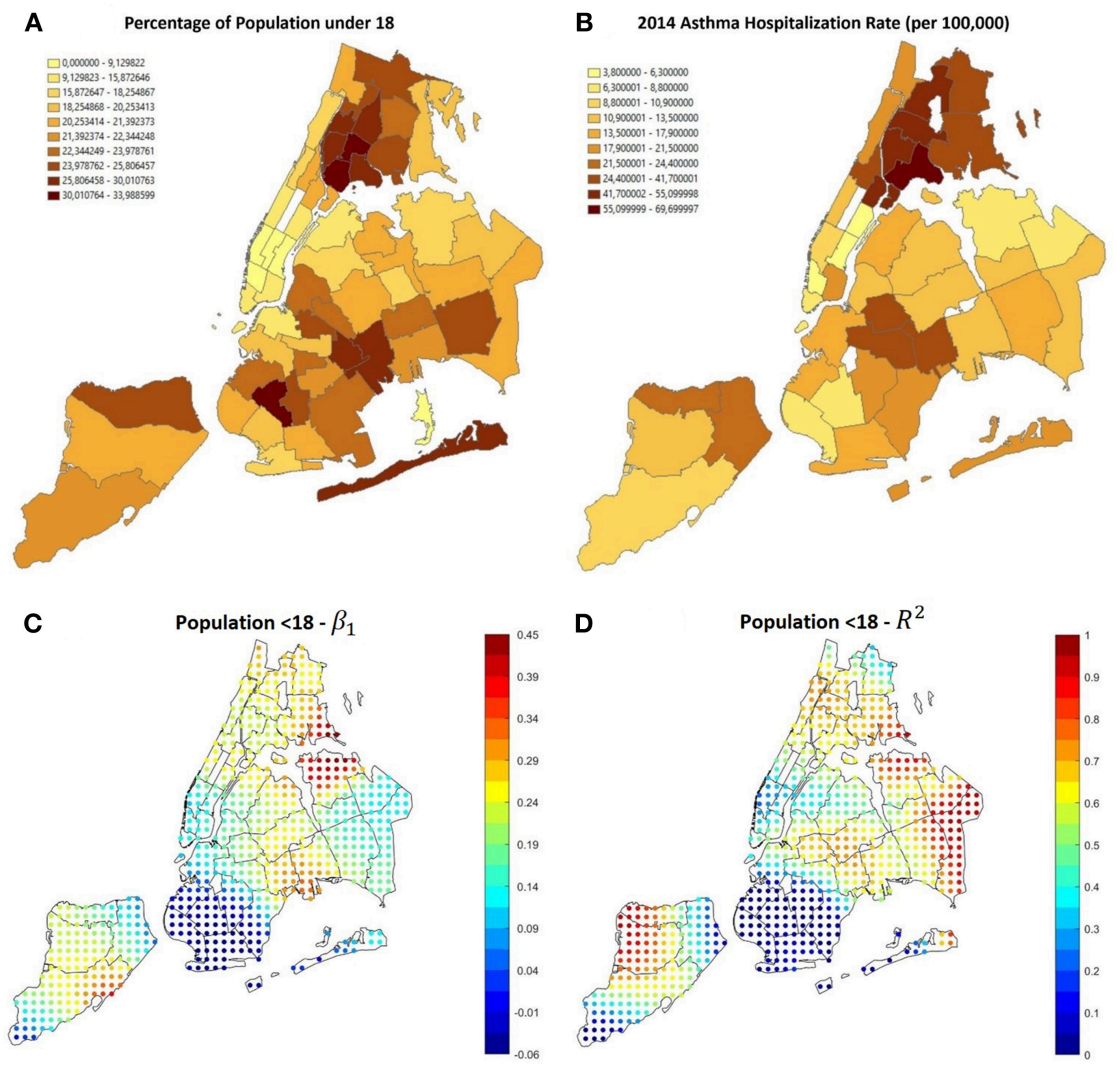
**(A)** Percentage of population aged under 18, most of it is concentrated in the Bronx and central-east Brooklyn, where also the asthma hospitalization rate is higher, as visible in **(B)**. **(C,D)** Results of GWR using the percentage of population aged under 18 as covariate. The correlation is positive and reliable in most of the areas with higher hospitalization rate.

**Figure 6:**
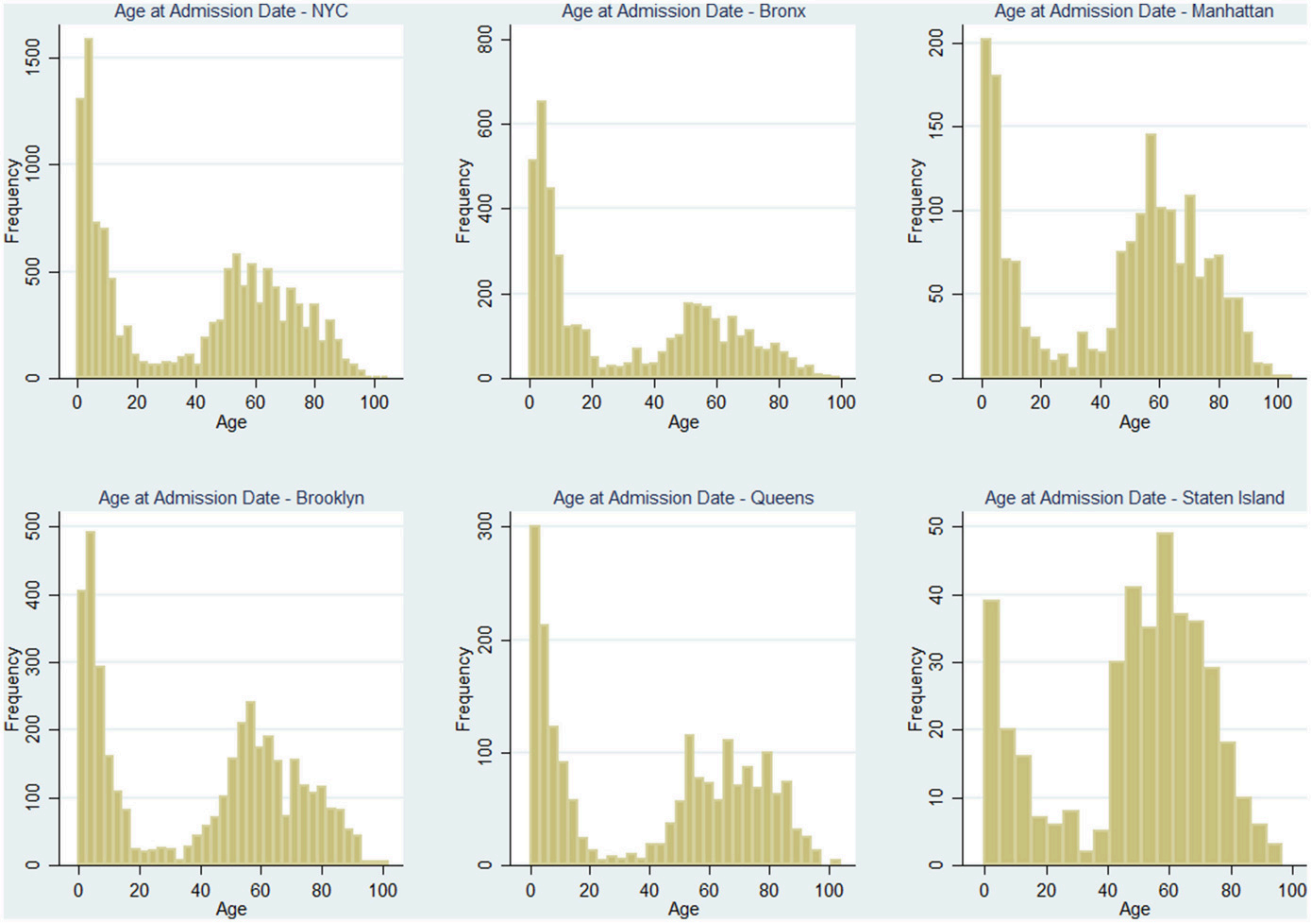
Distributions of patients' age at the moment of hospitalization through the year 2014, for the whole city and the 5 Boroughs separately.

It has been previously demonstrated that age is linked with the risk of asthma, as children and teenagers are more likely to suffer from it (30). Since services and cost of living vary throughout the city, the population age distribution changes among the different neighborhoods in NYC: as shown in Figure 5, population aged under 18 tends to concentrate in the central areas of the Bronx and east Brooklyn/west Queens, whereas the highest rates of population aged more than 65 can be found in Manhattan, east Queens, peripheral areas of the Bronx and south-west Brooklyn. Furthermore, Figure 6 shows that the age distribution of patients hospitalized for asthma in 2014 has the same shape in all neighborhoods, with a primary peak in young age and a secondary peak after the age of 40. The average hospitalization age changes in all the boroughs (from 31 in the Bronx to 49 in Staten Island, see Table 3 for details), and after running a one-way ANOVA test, we found that all age differences between boroughs were statistically significant (*P*-Value < 0.05) apart from the ones between Manhattan and Staten Island and Brooklyn and Queens. Therefore, age at hospitalization is significantly lower in the Bronx than in all the other boroughs, as it is lower in Brooklyn and Queens if compared to Manhattan and Staten Island. This is an interesting finding considering that the age-adjusted asthma prevalence per 100 individuals is 6.2 in the Bronx, 3.8 in Brooklyn, 4.6 in Manhattan, 3.7 in Queens and 5.7 in Staten Island (31) (data of the year 2002), therefore the areas with the higher prevalence are not the same with the higher hospitalization rates.

**Table 3:** Age at the moment of admission in NYC and in each borough.

**Borough**	**Average age**	**Standard deviation**	**Observations**
Bronx	30.85	28.24	4289
Brooklyn	39.88	30.14	3927
Manhattan	44.92	29.39	1832
Queens	41.45	32.22	1954
Staten Island	48.55	24.92	397
All	38.03	30.09	12399

**Figure 7:**
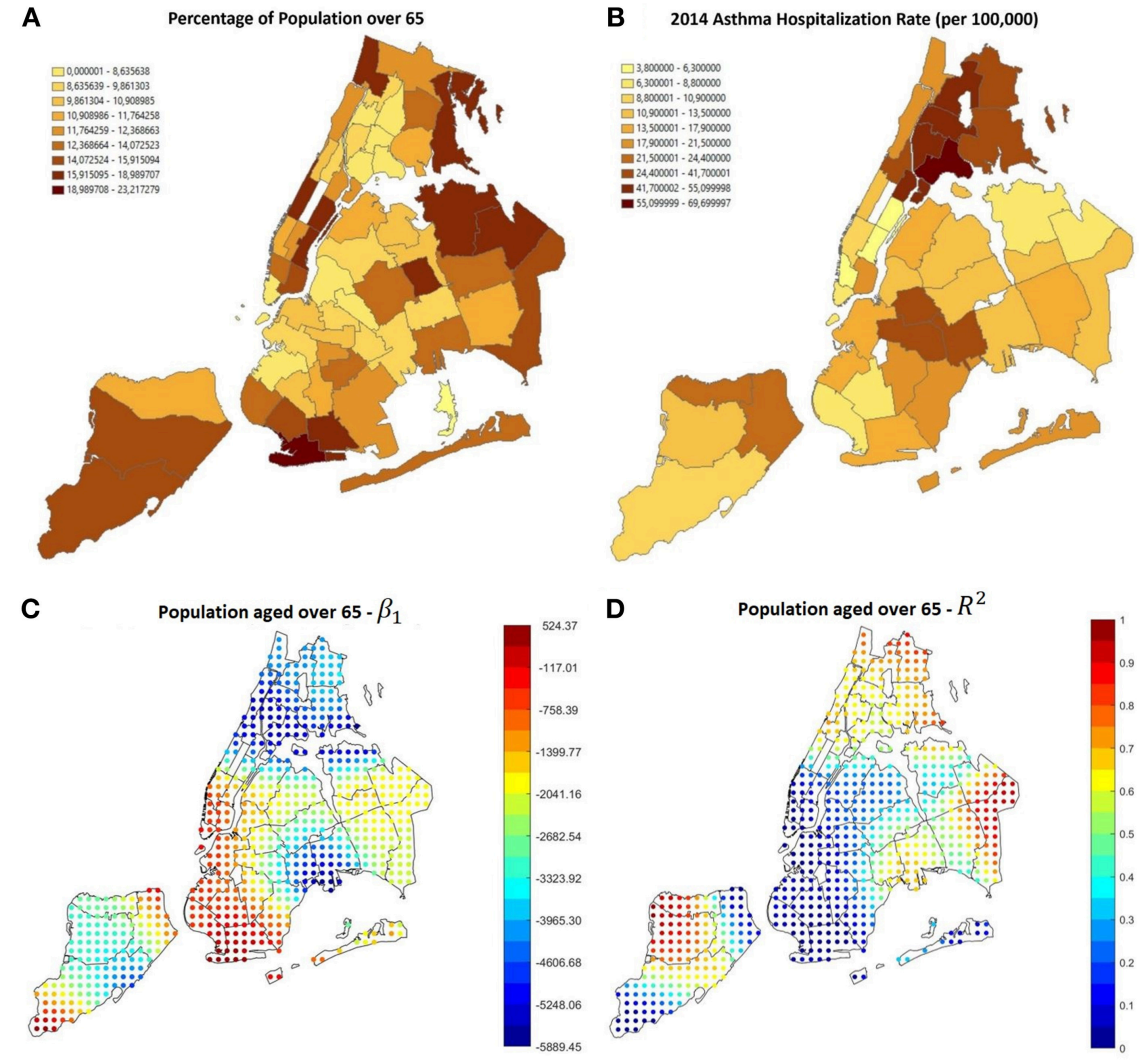
**(A)** Percentage of population aged over 65, most of it is concentrated in Manhattan, Staten island, East Queens and South-west Brooklyn, where the asthma hospitalization rate is lower, as visible in **(B)**. **(C,D)** Results of GWR using the percentage of population aged over 65 as covariate. The correlation is negative and reliable in most of the areas with low hospitalization rate.

Figures 5C,D, shows that GWR applied to percentage of people aged under 18 (Correlation 0.6389, *P*-Value 5.27 × 10^−6^) shows that in the areas with the higher prevalence and hospitalizations rate (i.e., the Bronx and east Brooklyn/west Queens), β_1_ is positive and *R*^2^ is high, this happens also in central Staten Island; using the percentage of population over 65 as covariate (Correlation−0.5342, *P*-Value 2.69 × 10^−4^) it can be noticed that in the same areas β_1_ is negative, therefore high rates of older people prevent hospitalization rates from rising (Figure 7).

### Health Insurance Coverage

**Figure 8:**
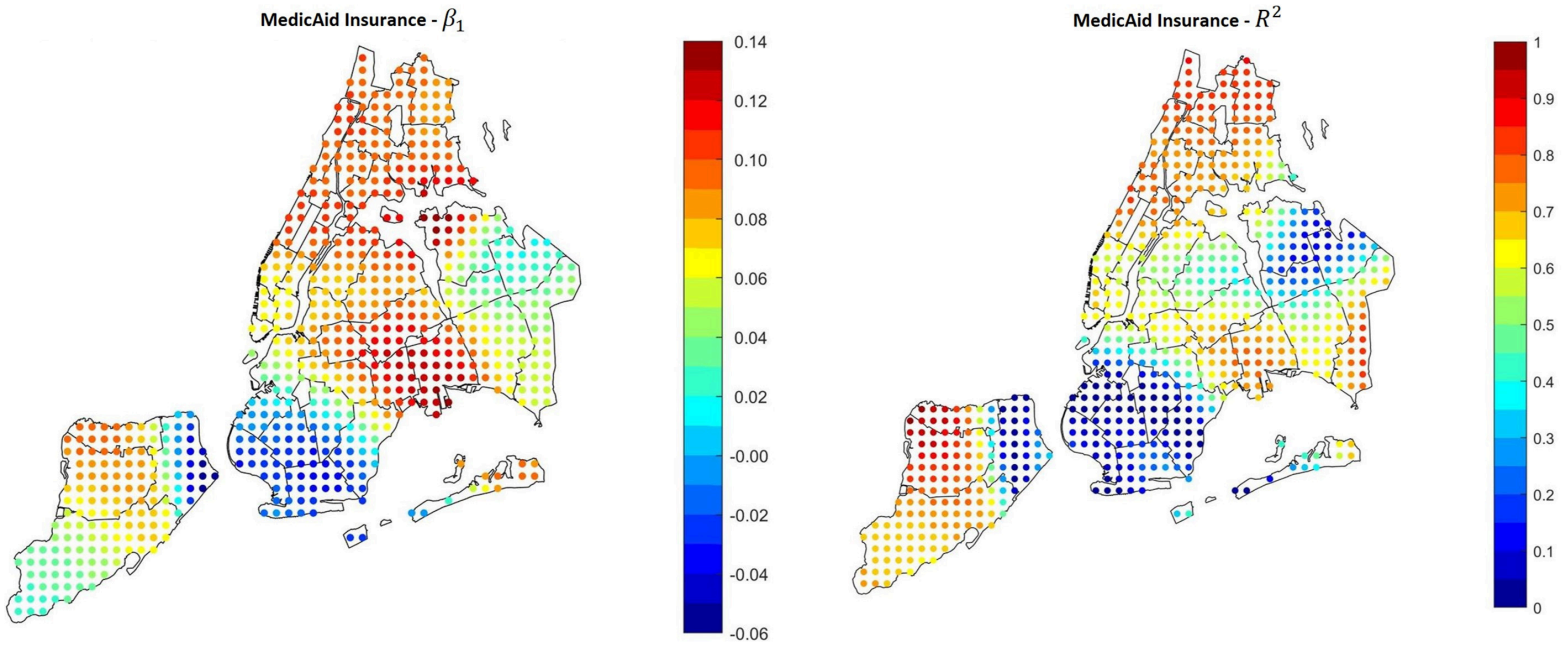
Results of the GWR applied using Medicaid coverage as covariate. In the areas with the higher hospitalization rate, the correlation is positive and significant.

The exploratory analysis through spatial clustering showed that there is a connection between lack of health insurance and hospitalizations. In addition, several studies demonstrated that people with no insurance or with public insurances such as Medicaid and Medicare tend to visit the ED more often that people with private insurance (26, 32). Our analysis confirmed that even insurance coverage can be a predictor for asthma hospitalizations in the high-rate areas of NYC: Figure 8 shows the results of the regression using the percentage of people covered by Medicaid as covariate (Correlation 0.7138, *P*-Value 11.1 × 10^−7^). The areas with the higher hospitalizations rate are the same with the higher Medicaid coverage, and also the same with the higher β_1_ and *R*^2^.

### Other Social and Environmental Factors

**Figure 9:**
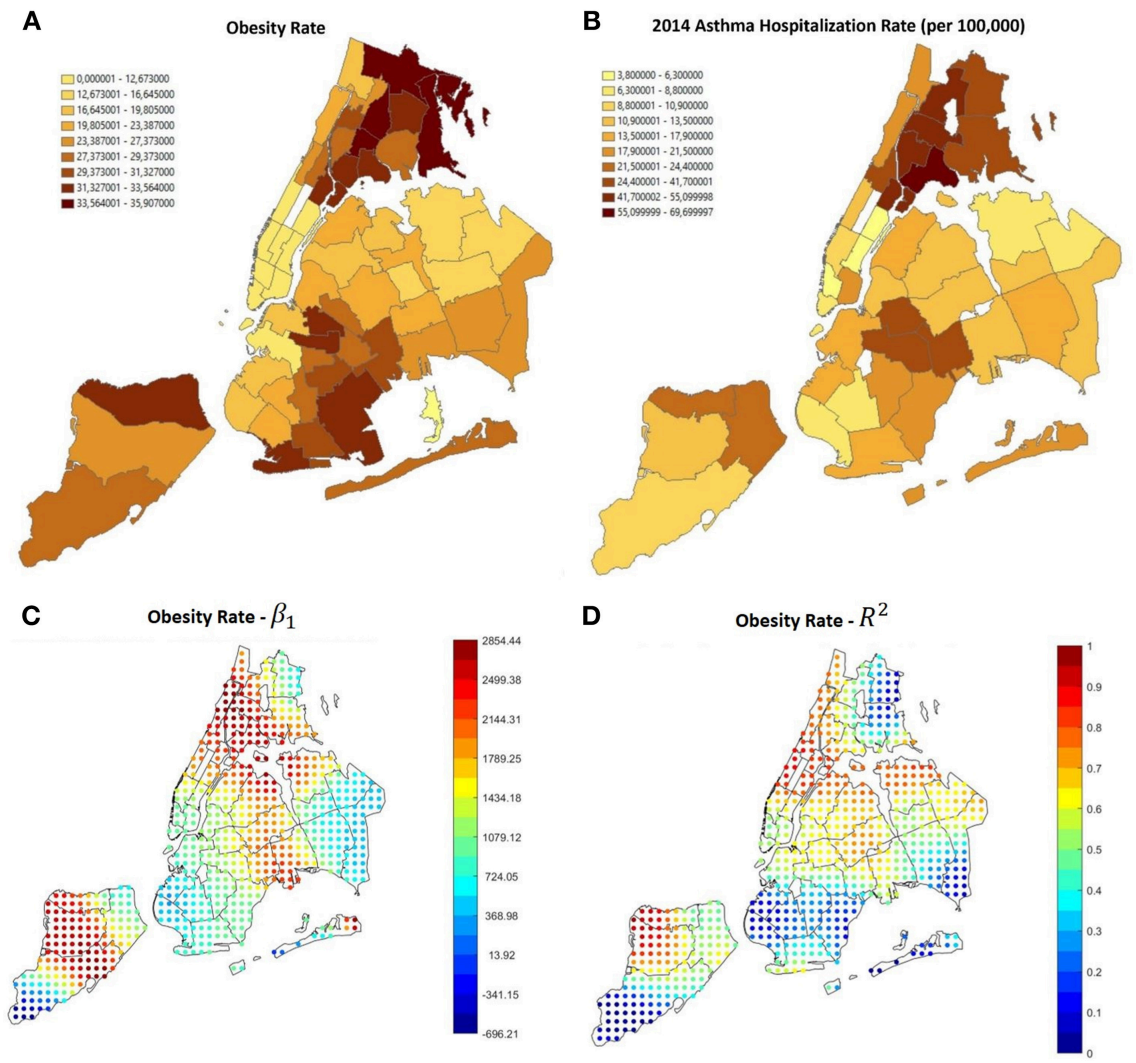
**(A)** Obesity rate in NYC, it is noticeable that the higher rates are in the same areas in which the asthma hospitalization rate is higher **(B)**. **(C,D)** GWR results using obesity as a covariate. The relation is generally positive.

In order to make our study as comprehensive as possible, we tested some other parameters. There are many other social and environmental factors that have been found to have an effect on asthma in previous research, one of them is obesity (33). Figure 9 shows that the higher obesity rates in NYC are between the Bronx, Upper Manhattan and East Harlem, south-east Brooklyn and north Staten Island. According to the GWS results there is a positive relation between obesity and hospitalizations in all the city, with high significance value in Upper Manhattan (especially East Harlem), Queens/east Brooklyn and north-west Staten Island. The overall correlation coefficient is 0.679 and the F-Statistic *P*-Value is 7.69 × 10^−7^.

**Figure 10:**
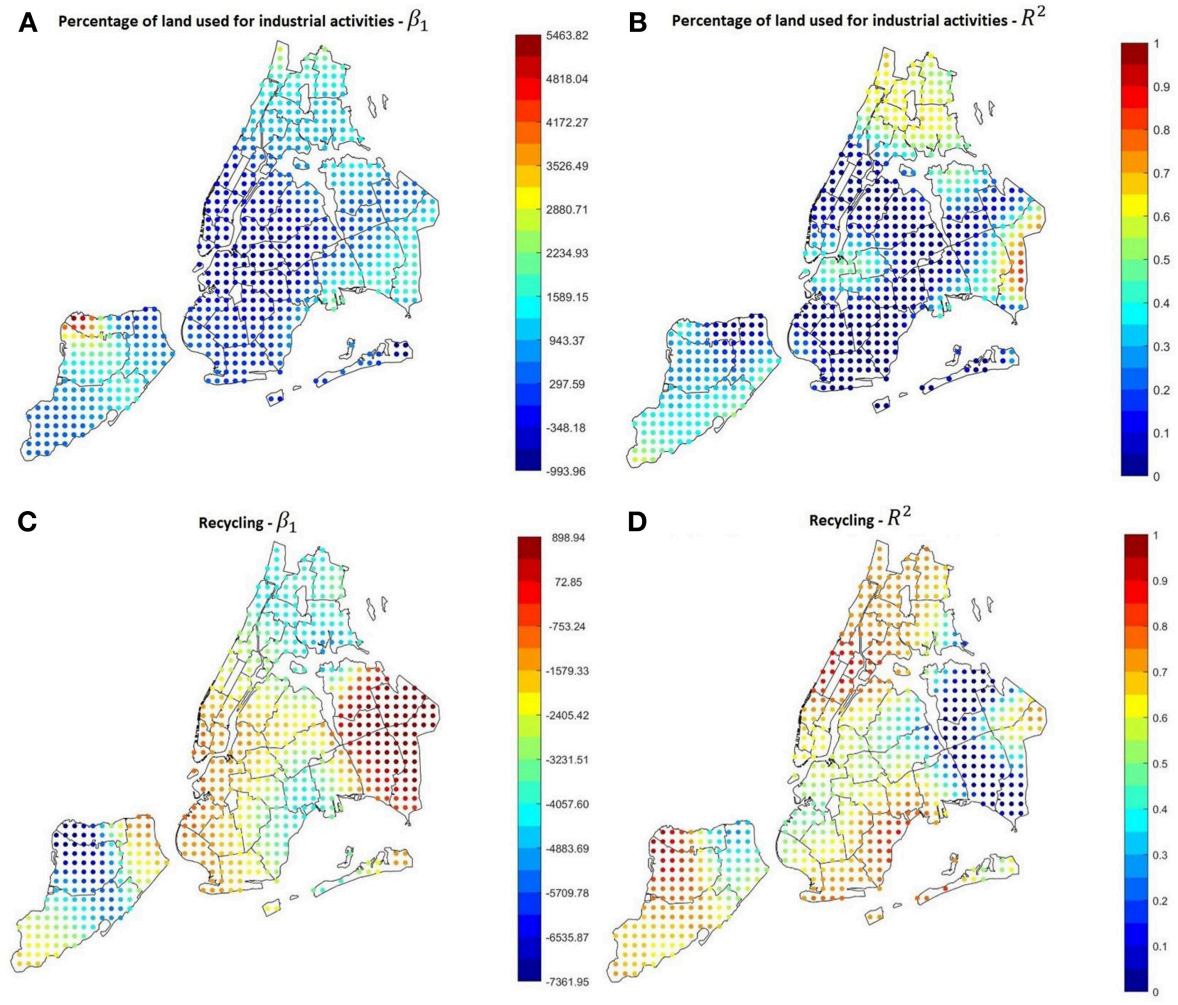
**(A,B)** GWR results using the percentage of land used for industrial activities as covariate. The relation is quite strong and positive in the Bronx. **(C,D)** GWR results using the percentage of recycled garbage as covariate. Excluding some areas in East Queens, the relation is quite strong and negative throughout the city.

Among other environmental factors that may influence asthma outcomes, we tested also the percentage of land used for industrial activities, and found, as shown in Figure 10, that there is a positive significant relation in the Bronx, where the hospitalization rate is higher, and in some other spots in Brooklyn and Queens. This could lead to the assumption that even if in a long-term measurement air pollution is higher in other neighborhoods, like in Manhattan, the presence of a lot of industrial sites could provoke brief local pollution peaks that could be a threat for people with asthma, this topic requires further investigation.

Another parameter that was surprisingly found to be correlated with the hospitalization rate is the garbage recycling rate (Correlation −0.7374, *P*-Value 2.55 × 10^−8^). In most of the city, especially the areas with the highest hospitalization rates, higher recycling rates correspond to lower asthma hospitalization rates, as shown in Figures 10C,D.

### Multivariate Analysis

GWR can be also performed testing several covariates at the same time, creating a multivariate model. The advantage of this kind of model is the ease of comparison of the effects of different variables, that allows to spot outliers, confounders and relations between different factors. Since the univariate results, as well as several studies published in literature, highlighted how poverty rate and race play an important role in increasing the probability to get hospitalized for asthma, we created an example of multivariate GWR that combines poverty rate and percentage of people identifying as Black and Hispanic. The underlying model can be described by the following equation, valid for each point where the GWR is performed:

$$\begin{array}{l} Hospitalizations=\;\beta_{0}+\beta_{1}*Poverty+\beta_{2}*\%Black\\ \;+\beta_{3}*\%Hispanic \end{array}$$

**Figure 11:**
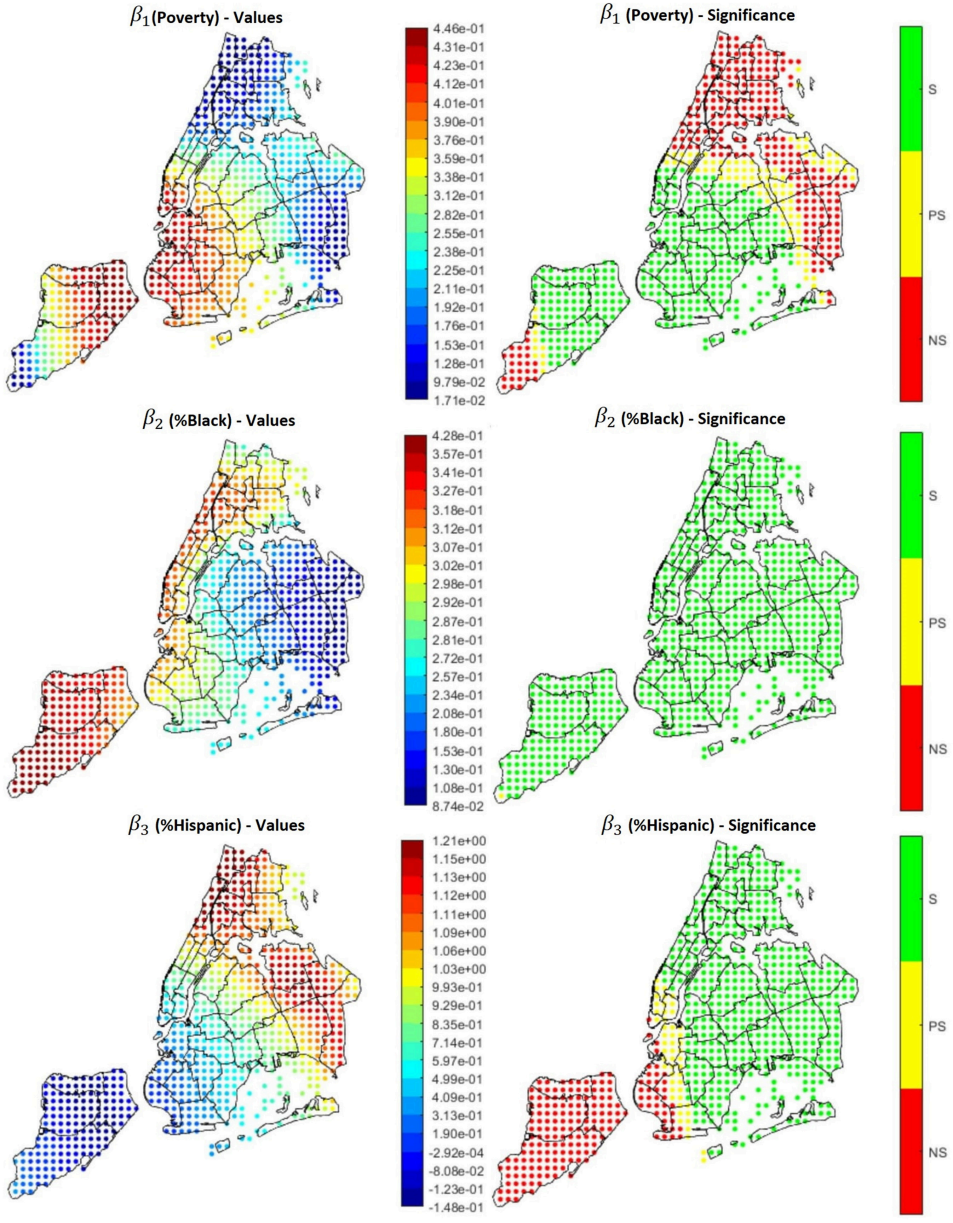
Coefficients (left side) and their significance level (right side) for the multivariate model Hospitalizations = β_0_ + β_1_*Poverty + β_2_*%Black + β_3_*% Hispanic.

**Figure 12:**
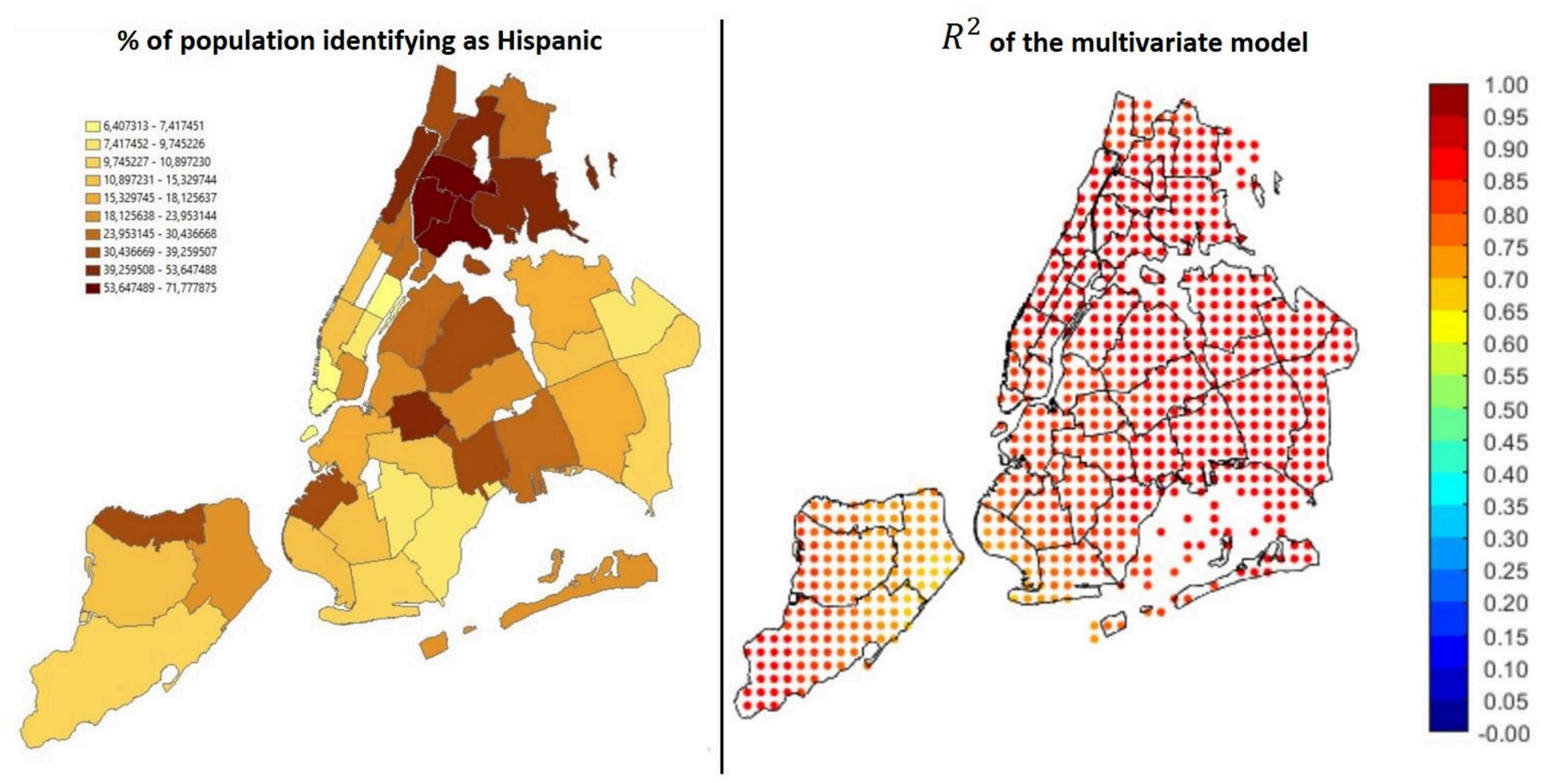
On the left: percentage of Hispanics over the total population. On the right: global *R*^2^ of the model.

Results are visible in Figure 11. On the left side of the image, maps of the β coefficients are shown, whereas panels in the right side show the correspondent significance maps based on the t-statistic values. In detail, we created 3 significance levels: Non-Significant (NS), Partially Significant (PS), Significant (S). The correspondent t-statistic threshold values are 1.96 (5% confidence level) and 2.58 (1% confidence level). Figure 12 shows the percentage of Hispanic people in the different neighborhoods on the left side (useful for the analysis reported below in this section) and the global *R*^2^ of the model. Several interesting phenomena can be noticed in these figures:

- *R*^2^ is extremely high in all the region, therefore the linear model is globally reliable;
- Considering poverty and percentage of Black population, the correspondent β are always positive, indicating a positive correlation between either of these factors and the hospitalization rate;
- In general, the higher the β, the higher the level of significance. Therefore, in the neighborhoods in which we found that high variables' levels lead to high hospitalization rates, the found relations are significant;
- Low significance levels could be due to the effect of other confounding variables and to a smaller quantity of data available. For instance, Figure 12, left side, shows the values of the variable associated to β_3_, i.e., percentage of Hispanic people. It can be noticed that most of the Hispanic population is concentrated in the Bronx, Upper Manhattan (Harlem, East Harlem and Washington Heights), in central and west Queens and some areas of east Brooklyn (Bushwick and south of Highland Park), plus some isolated spots in west Brooklyn (Sunset Park) and north Staten Island. Apart from these last isolated spots, in the same areas in which the concentration is higher, also the significance of the correspondent beta is high. Hence lower significance corresponds to higher scarcity of data.

These results show that even multivariate geographical analysis can be helpful to describe and visualize important public health phenomena and discover the relations among different factors.

## Discussion

The relation between air quality and respiratory diseases such as asthma is a widely studied topic in the medical literature. Despite the strength of the evidence connecting specific pollutants to new-onset asthma is often variable (34), Toskala (4) analyzed risk factors associated with its development and found that the disease is often the result of a complex combination of genetic predisposition and dysfunctional interaction with the environment to which we are exposed. On the other hand, a recent review from Guarnieri (14) pointed out how previous research almost unequivocally connects pre-existing asthma exacerbation episodes to air quality and pollution. Among the most relevant environmental factors is small particulate matters, which cause oxidative injury to the airways, leading to inflammation, remodeling, and increased risk of sensitization. Several studies have addressed the specific effect of PM 2.5 exposure on the human respiratory system, especially in heavily polluted areas like metropolitan regions of China (35), where PM 2.5 has been shown to have an adverse impact on asthma ED visits even after short-term exposure (36). Kloog (12) also investigated the dual effect of long- and short-term exposure to small particulate matters on asthma-related deaths in Massachusetts, reporting their positive correlation with mortality.

Despite its known link with air pollution, asthma remains a multifactorial disease whose outcomes depend on a multitude of social and environmental phenomena. A big city is the most suitable environment to study the cause-effect dynamics that lead to asthma complications. Several studies concerning urban areas have been carried out in the past, but they mostly focus on large geographic areas. Studying asthma hospitalizations at a finer spatial resolution can help to inform policy makers and citizen themselves on the situation in the different neighborhoods, in order to organize targeted interventions and prevent new hospitalizations from happening as much as possible. The PULSE project is founded on this principle and aims at spreading it to all the world's biggest cities.

In this preliminary study, we were able to show the connections between asthma and several environmental and social factors in New York City. Asthma treatment is a common topic in NYC, since the New York State healthcare system spends 1.3 Billion dollars per year for asthma (second highest state in the US) and current asthma prevalence in adults living in New York City is 10.2%, higher than 9.3% of adults who live in the rest of the State (13). Our results show that socioeconomic factors play a fundamental role in determining the hospitalizations rate. In particular, according to our results a higher risk of asthma is associated with poverty, race/ethnicity, age (risk increases in younger patients), obesity, proximity to industrial areas, proximity to low recycling areas. We found that poor people and people without insurance or covered by Medicaid are more likely to visit the hospital for asthma, in accordance with previous studies that showed a different use of the hospitals from people with different kinds of insurance, demonstrating that people without insurance or with public programs such as Medicaid are more likely to visit ERs. We then found that the age at hospitalizations is lower in the Bronx compared to the other areas of the city, most of the highest peaks of hospitalization rate are in this borough too, as well as a generally higher poverty rate. In a limited part of the city that includes south-West Brooklyn and East Staten Island, some factors such as poverty, obesity and insurance coverage have little or no effect on the hospitalizations rate. Deepening the analysis, we are able to hypothesize that this is due to particular conditions of the environment (i.e., pollution is low, there are no factories, garbage disposal is adequate) and of the population (medium income and generally adult) that prevent hospitalizations from happening. Further investigation in these areas is indeed required, nevertheless this demonstrates that spatial enablement is necessary to aid public health in big cities, providing useful tools both for visualization and discovery.

We found a weak link between asthma and average PM2.5 in 4 out of 5 boroughs, this demonstrates that air pollution alone doesn't influence significantly the asthma hospitalization probability, we also investigated the relation between ozone concentration and asthma, but results were unreliable. Further investigation on the link between pollution and asthma hospitalizations may be required, since yearly averaged data could be unsuitable to spot the real correlations, as local and temporary peaks are not visible. Furthermore, asthma is known to be related to different kinds of pollutants (14), which can exacerbate the disease through a combined effect that sometimes is difficult to detect. This is due also to a frequent lack of pollution data with a sufficient granularity in space and time. In New York City, for instance, there are only 13 official monitoring stations over a 784 km^2^ area, and not all of them measure the same pollutants. High-quality air monitoring stations are expensive, unable to identify pollution hotspots and require a lot of maintenance (37), whereas small and relatively cheap sensors are sometimes unreliable. Within PULSE, a high number of small and portable sensors are being deployed in each city, allowing to generate a dense sensor network and collect high-resolution measurements to be used in future analyses regarding air quality.

Asthma exacerbation has indeed also some temporal/seasonal features, since it has been demonstrated to be correlated with high diffusion of respiratory viruses, common in the cold season, and with high pollen concentration in the spring period. It is also known that several of these factors have a different impact on pediatric asthma rather than adult asthma. All these facts should be addressed to take into account all the different characters of this disease, unfortunately availability of open data regarding these topics, especially referenced to a granular geographic subdivision, is limited.

This situation limits also the usability of spatially enabled model as the ones presented in this paper, in particular the multivariate linear regression, since the small number of observations (42 in our cases, correspondent to the 42 UHF polygons) doesn't allow the inclusion of a large number of covariates, leading to a possible bias by endogeneity considering the possible presence of correlation between the covariates and the error term (that depends on all the other variables not included in the model). This model can be therefore better exploited using more data collected in a more granular spatial description.

Thanks to the comprehensive architecture that the PULSE consortium is developing, a lot of data is being collected from several cities, and more analyses such as the one presented in this paper will be possible with more recent information and even finer spatial and temporal resolution, and both citizens and public policy makers will be directly involved. The large amount of new data collected and the high spatial resolution (some of the data collected so far is currently describable at a zip code level in NYC, i.e., with 264 polygons) will allow to extend the methods described in this paper creating more comprehensive and precise models.

One of the final steps of PULSE is the establishment of the Public Health Observatories (PHOs), that will assist public health policy makers in the decision-making process. Hospitalizations are an easy parameter to monitor, and knowing in which areas their rate is higher and which factors determine their occurrence can be useful to plan targeted interventions and design new public policies that can improve health and well-being, reducing also healthcare costs. Thanks to the PULSE system, urban health statistical analyses are carried out at a neighborhood level, and population is stratified according to a combination of environmental exposure and general features, allowing to understand which population categories are more at risk and to optimize the health assistance services according to a precision medicine paradigm. Regarding the specific links between asthma and risk factors found in this paper, it cannot be neglected that some of the factors that have been addressed are manageable more easily than others by public health authorities, for example traffic reduction and car improvements laws can limit air pollution, delocalization of industrial activities from the city, combined with an increase of the number and the extension of green areas can improve the environment, food policies and sensibilization campaigns can reduce obesity etc. On the other hand, it is quite difficult for a local authority to intervene on factors such as poverty and insurance, that are related to central health policies and rules. Nevertheless, our findings demonstrate the utility of our approach and provide an example of the importance of using highly spatially-enabled techniques to address health problems in urban environments.

## Author Contributions

All the authors contributed to this work and approved the final version for submission. DP and EP wrote the paper. DP, VC, and MR carried out the analyses reported in this study. JP granted access to the data and helped informing the discussion. RB and VC overviewed the study and led the work packages of PULSE regarding the geo-enabled components.

### Conflict of Interest Statement

The authors declare that the research was conducted in the absence of any commercial or financial relationships that could be construed as a potential conflict of interest.
